# Changes in Heart Rate Variability Are Associated with Expression of Short-Term and Long-Term Contextual and Cued Fear Memories

**DOI:** 10.1371/journal.pone.0063590

**Published:** 2013-05-07

**Authors:** Jun Liu, Wei Wei, Hui Kuang, Fang Zhao, Joe Z. Tsien

**Affiliations:** 1 Key Laboratory of Brain Functional Genomics (Ministry of Education), Institute of Brain Functional Genomics, East China Normal University, Shanghai, China; 2 Banna Biomedical Research Institute, Xishuangbanna, Yunnan, China; 3 Brain and Behavior Discovery Institute and Department of Neurology, Medical College of Georgia, Georgia Regents University, Augusta, Georgia, United States of America; CSIC-Univ Miguel Hernandez, Spain

## Abstract

Heart physiology is a highly useful indicator for measuring not only physical states, but also emotional changes in animals. Yet changes of heart rate variability during fear conditioning have not been systematically studied in mice. Here, we investigated changes in heart rate and heart rate variability in both short-term and long-term contextual and cued fear conditioning. We found that while fear conditioning could increase heart rate, the most significant change was the reduction in heart rate variability which could be further divided into two distinct stages: a highly rhythmic phase (stage-I) and a more variable phase (stage-II). We showed that the time duration of the stage-I rhythmic phase were sensitive enough to reflect the transition from short-term to long-term fear memories. Moreover, it could also detect fear extinction effect during the repeated tone recall. These results suggest that heart rate variability is a valuable physiological indicator for sensitively measuring the consolidation and expression of fear memories in mice.

## Introduction

Fear conditioning is among the most widely used associative learning and memory tests, especially in laboratory animals such as mice and rats [1]–[3]. The two major forms of fear conditioning protocols are contextual fear conditioning and cued fear conditioning. In a typical experimental paradigm, the presentations of a conditioned stimulus (CS), such as a neutral tone, are coupled with an unconditioned stimulus (US), such as a mild foot-shock in shock chamber [2], [4]–[6]. With repetitions of paired CS-US presentations in a given environment, animals learn to fear the conditioned tone (cued fear conditioning) or the context in which the animals were conditioned (contextual fear conditioning). There are several variations in terms of the nature of conditioned (tone, light, odor, environment, etc.) and unconditioned stimuli (mild electrical foot-shock, air-puff to eyelid, etc.). Nonetheless, these fear conditioning protocols can produce long-lasting associative memories in the brains of various animal species, ranging from flies to mice to humans. It has been shown that contextual fear conditioning is hippocampal dependent, whereas cued fear conditioning is hippocampal independent [1], [2], [7]. Both types of fear conditioning require the activation of the NMDA receptors [8]–[12].

In mice and rats, acquisition of fear conditioning memories is typically measured by increased freezing behavior which is defined as the absence of body movement except for breathing [13], [14]. The freezing measurement is discrete in the time domain, and usually calculated as a percentage of the total given time. For cued fear conditioning, the fear memory is typically measured in a different chamber or box distinct from the shock chamber. This is largely because that mice or rats in the home environments may stay immobile which can compound the freezing measurement. Thus, fear memory retrieval in home cage environments by conditioned cue was usually not studied. To expand behavioral readout of fear memories, measurements of heart rate, blood pressure, breathing, or muscle activity have been reported in combination with freezing scoring [15]–[19]. More recently, we have used our large-scale *in vivo* recording technique and successfully measured real-time memory traces of fear memories in the CA1 region of the mouse hippocampus [20]. Our neural decoding approach has revealed that CA1 produced many forms of distinct CS and US memory traces, including memory traces for predicting the CS-US time interval [20].

Yet we have also found that not all real-time memory traces revealed in our decoding algorithms fall into categories of CS or US memory traces. We believe that this could be due to limited classifications of experimental or physiological parameters for peri-event spike histogram analysis (i.e. CS, US, or freezing and unfreezing, etc.). Therefore, there is a strong need to provide additional characterization of fear conditioned-induced physiological changes such as heart rate (HR) and heart rate variability (HRV) changes during the various stages of fear memory formation. Such detailed knowledge can provide highly useful physiological or emotional markers for aiding the identification of novel components of emotional memories in memory circuits.

Therefore, in this present study, we set out to systematically characterize HR dynamics on fear learning and their temporal evolution over multiple CS-US pairing trials. Moreover, we further investigated HR and HRV changes associated with short-term memory and long-term memory retention stages. We found that fear conditioning produced robust short-term and long-term fear memories that were invariantly characterized by two temporal stages marked by distinct HRV. The durations of these two distinct HRV stages should be useful to provide more refined neurophysiological description of fear and emotional memories.

## Results

### ECG recording during different behavioral states in freely behaving mice

**Figure 1:**
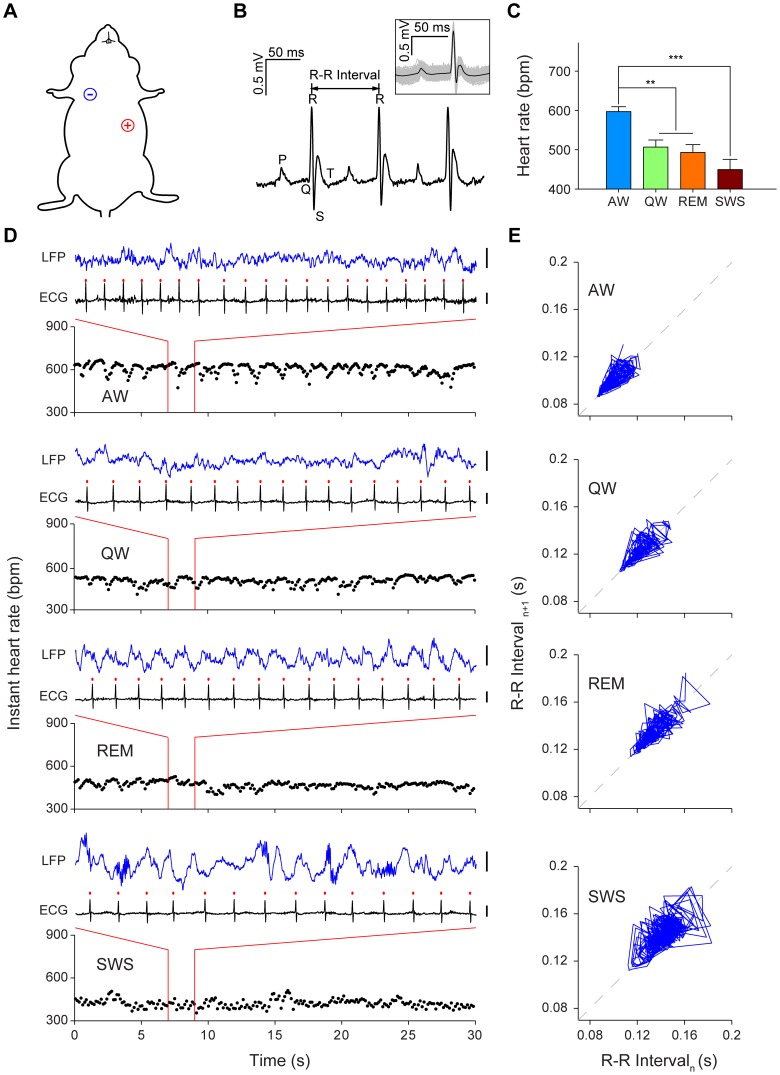
ECG recording during four behavioral states. (A) Illustration of the proper ECG electrodes implantation sites on a mouse. The negative electrode (-) was implanted in the mouse’s right upper chest, and the positive electrode (+) was placed in the left abdomen. (B) Three consecutive cycles of heart beats as example of ECG recording. The peaks are labeled by conventional ECG terminology. Inset: average waveform of individual heart beats recorded in 1 minute, centered on the peak of the R-wave. (C) Mean heart rate of mice during four basic behavioral states in the home cage. AW, active wakefulness; QW, quiet wakefulness; REM, rapid eye movement sleep; SWS, slow wave sleep. Error bars, s.e.m.; n = 5; **P<0.01, ***P<0.001, one-way repeated measures ANOVA and Tukey *post hoc* test. (D) Examples of 30-sec instant HR and 2-sec ECG and hippocampal CA1 LFPs during AW, QW, REM and SWS from an individual mouse. The red dots indicate the peaks of the R-wave. Scales: 0.5 mV. (E) Poincaré plot analysis graphed the same mouse’s R-R interval data of four 1-min periods: during AW, QW, REM and SWS. Successive points in the plots were connected with a line.

We implanted a single pair of electrodes into mouse’s right upper chest and left abdomen to allow recording of electrocardiogram (ECG) signals (Figure 1A). Data from 11 mice were included in the current analyses. The typical peaks in heart beats could be clearly identified with the timestamps of R-wave peaks (Figure 1B). To provide the overall characterization of HR dynamics in mice, we employed hippocampal local field potentials (LFPs) to classify mouse behavior into four basic states in the home cage: namely, active wakefulness (AW), quiet wakefulness (QW), rapid eye movement (REM) sleep and slow wave sleep (SWS). We calculated the mean HR during these states and found averaged HR as follows: 597±12 bpm for AW, 507±18 bpm for QW, 493±20 bpm for REM, and 449±26 bpm for SWS (Figure 1C). The HR during AW was significantly higher than during QW, REM and SWS states (Figure 1C, *P*<0.01, *P*<0.001). The HR during QW, REM and SWS showed no significant difference between each other (Figure 1C, QW:REM, *P* = 0.956; QW:SWS, *P* = 0.098; REM:SWS, *P* = 0.215).

In order to measure the real-time HR dynamics, we used the instant HR, which was derived from R-R intervals. During these four different behavioral states, the instant HR revealed different HR dynamics with noticeable HRV from beat to beat (Figure 1D). In order to provide quantitative measurement of real-time HRV, we employed Poincaré plot to analyze the data. Poincaré plot is a nonlinear dynamic technique which can be used to quantitatively visualize the fluctuation in the R-R intervals [21], [22]. Our Poincaré plot analysis revealed distinct HR and HRV for each state (Figure 1E). For example, active wakefulness (when mouse was walking or grooming in its home cage) exhibited both higher HR and reduced HRV in comparison to those of slow wave sleep. The positions of cluster and the dispersion of data points for AW were located in the lower left corner, whereas SWS data was more spread on the upper right corner of the plot (Figure 1E).

### Heart rate variability is highly sensitive to handling and novel environment exposure

**Figure 2:**
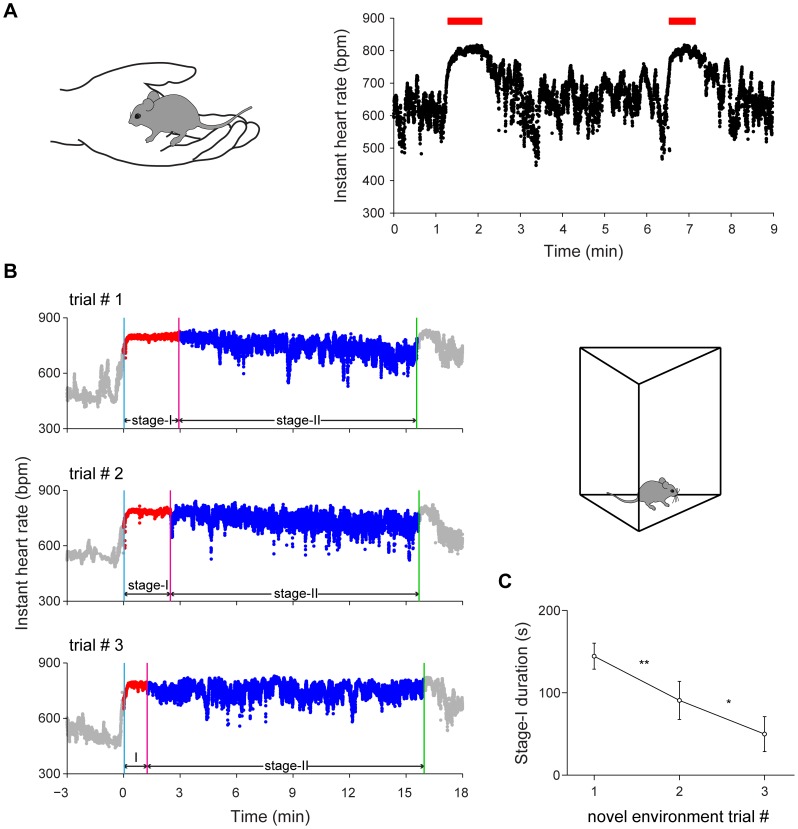
Instant heart rate responses to handling and novel environment exposures. (A) Illustration of the handling method: cup handling [38] (left) and two examples of instant HR response to handling (right). The red bars indicate the duration of handling (49 seconds and 37 seconds, respectively). (B) Instant HR responses of an individual mouse during repeated exposures to a novel environment (left panels) and illustration of a novel environment (right). The blue vertical line indicates the start of stage-I; the red vertical line indicates the end of stage-I; the green vertical line indicates the end of the novel environment exposure. (C) The duration of stage-I during repeated novel environment exposures. Error bars, s.e.m.; n = 7; *P<0.05, **P<0.01, one-way repeated measures ANOVA and Tukey *post hoc* test.

Typically, prior to fear conditioning experiments, mice are picked up from home cages and then placed into a conditioning chamber. This standard practice can represent a dramatic change in physical and emotional states for animals and was usually repeated prior to fear conditioning as a habituation procedure. We asked how heart rate (HR) and heart rate variability (HRV) might change by this handling procedure. We found robust increase in HR at the onset of picking-up the mice from the home cages by the experimenter (Figure 2A). We noticed that in addition to HR increase, placing mice in hand also caused a sustained reduction of HRV (red bar in Figure 2A). This HRV reduction phase was correlated with the duration of time during which mice stayed in hand (see examples in Fig. 2A, two holding periods: 49 seconds for the first epoch, and 37 seconds for the second epoch, respectively). However, once the mouse was placed back in the home cage, this reduced HRV phase quickly returned to the more variable base level. At the same time, HR would also reverse back to the base rate. This transient hand holding effect on HRV were consistently observed despite repeated handling of mice for many days (two sessions of 5 minutes handling each day for 5 days). This suggests that hand handling is a transient emotional state, correlated with the time duration of mice staying in experimenter’s hands.

Next, we investigated how a new environment distinct from its home cage might change emotional states of the mice. When mice were introduced to a novel environment, we noted that it increased HR and reduced HRV at the same time. Overall, HR increased from 579±29 bpm in home cages to 756±11 bpm in a novel chamber when the mice entered the novel chamber (Figure 2B, trial #1). Moreover, we found that HRV also exhibited dynamic changes, with the most notable reduction of HRV initially (stage-I) and followed by a more relaxed stage with larger HRV (stage-II) (Figure 2B). In order to distinguish these two stages qualitatively and quantitatively, we combined the threshold method with a sliding-window technique (see methods for details) to define the low HRV phase (stage-I in red) and the more variable phase (stage-II in blue). Interestingly, we found that the duration of stage-I was sensitive to habituation procedures. As shown in Figure 2B, the duration of stage-I notably decreased by repeated exposures (trial #2 and trial #3) to the same novel environment. Quantitative measurement of the durations of stage-I from trial #1 to trial #3 showed a statistically significant decrease (Figure 2C, *P*<0.01, *P*<0.05, respectively). Interestingly, we found that the HR did not show significant difference between different habituation sessions (*P*>0.05). This suggests that HRV, rather than HR itself, is a more sensitive parameter for reflecting real-time emotional updates in mice. This increased heartbeat regularity in the stage-I may represent a useful physiological signature reflecting novelty- or fear-triggered changes in emotionality.

### Changes in heart beat dynamics upon fear conditioning

**Figure 3:**
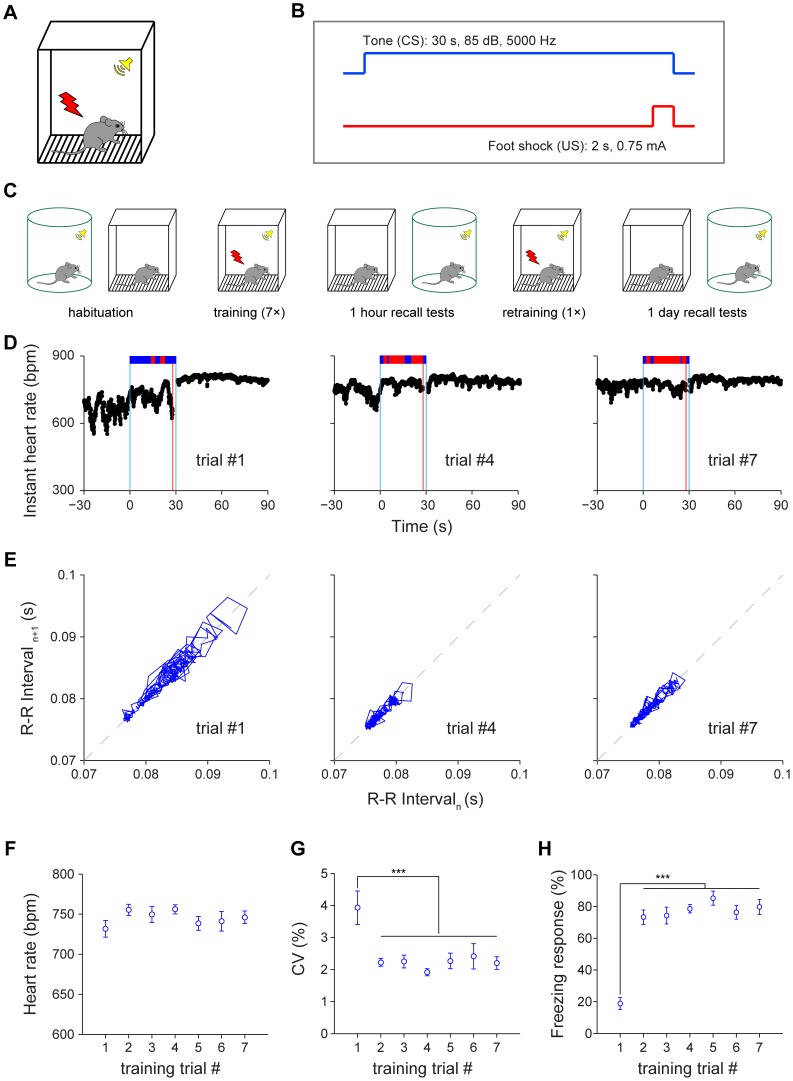
Effects of fear conditioning on heart rate and heart rate variability. (A) Illustration of a fear conditioning chamber for producing a tone-shock pairing fear memory. (B) A 30-sec neural tone (85 dB, 5000 Hz) co-terminates with a 2-sec mild foot shock (0.75 mA). (C) Schematic representation of experimental paradigm for cued fear conditioning. (D) Instant HR responses of an individual mouse during three CS-US pairings, trial#1, trial#4 and trial#7. The freezing responses were plotted on top of the instant HR; freezing state, red bar; non-freezing state, blue bar. The blue vertical lines indicate the onset and offset of the tone (30 seconds); the red vertical line indicates the onset of the foot shock (2 seconds). (E) Poincaré plots of the same mouse’s R-R intervals of 28-sec tone duration in trial#1, trial#4 and trial#7. (F) The average HR of 28-sec tone duration during training. (G) The average CV of instant HR of 28-sec tone duration during training. (F) The average freezing responses of 28-sec tone duration during training. Error bars, s.e.m.; n = 11; ***P<0.001, one-way repeated measures ANOVA and Tukey *post hoc* test.

To produce fear conditioning memories, we used classic conditioned stimulus (CS, 30-sec tone, 85 dB, 5000 Hz) which was co-terminated with a brief 2-sec unconditioned stimulus (US, foot shock, 0.75 mA) during the acquisition phase (Fig. 3A and 3B). To provide a detailed understanding of how fearful emotion may evolve during the learning phase, we used a seven CS/US-pairing protocol with 3–5 min randomized interval between each CS-US pairing. For measuring HR dynamics during recall, we performed contextual and cued retention tests at 1-hr (short-term memory) and 1-day (long-term memory) (Fig. 3C). To prevent potential fear extinction during short-term retention, the mice were subjected to one more CS/US pairing before being brought back to home cages for rest. The long-term fear memory was measured at 1-day intervals for both contextual and cued retention tests (24 hours later) (Fig. 3C).

Prior to the first CS/US pairing, we noticed that instant HR was at a higher state in the conditioning chamber (despite of habituation) in comparison to that of home cages, reflecting the effects of novel environment on mice. Upon CS/US pairing, HR exhibit small further increases and seemed to reach the ceiling effect. Yet the most prominent feature upon fear conditioning was the great reduction in HRV (Figure 3D). The Poincaré plot of R-R intervals during the tone period revealed interesting dynamic patterns in HRV decrease (Figure 3E). Although the average HR did not show additional significant change over the seven trials (Figure 3F, *P*>0.05), the coefficient of variation (CV) of instant HR, a measurement of HRV, did reveal a dramatic decrease beginning with the second trial of CS/US pairing (Figure 3G, *P*<0.001). Behaviorally, mice exhibited a significant increase in immediate freezing during tone right after the first pairing (Figure 3H, *P*<0.001). This indicates that HRV, rather than HR, is better at reflecting changes in dynamic emotional status of mice undergoing fear conditioning.

### Two distinct HRV stages during both 1-hr and 1-day contextual memory recall

**Figure 4:**
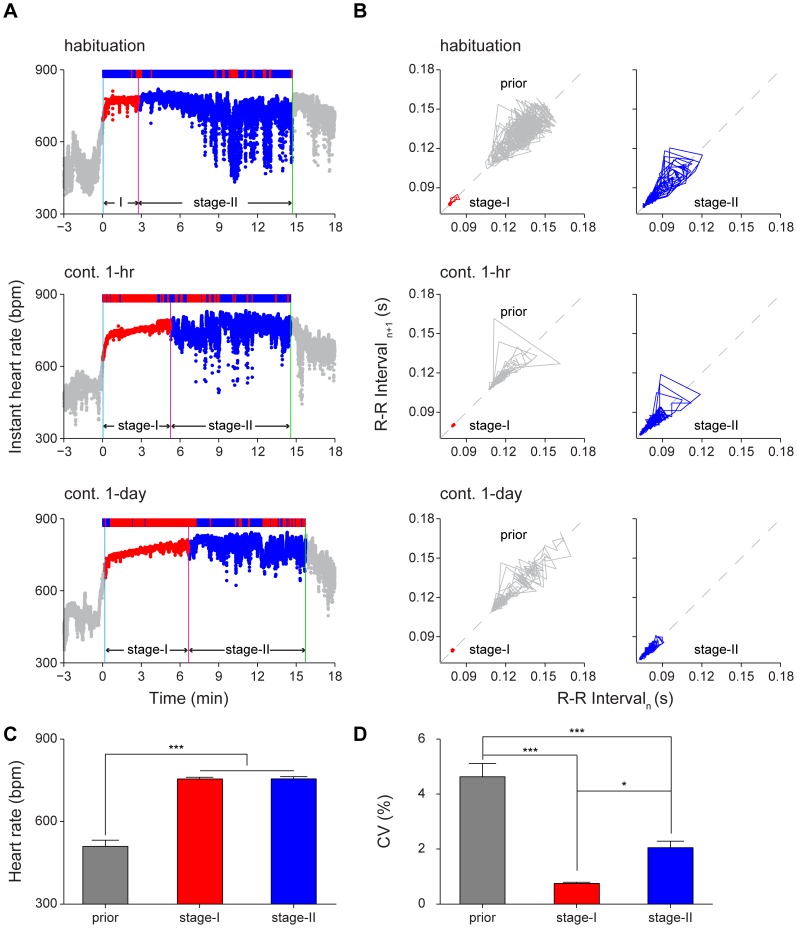
Increased duration of heart rate variability stage-I in long-term contextual memory retention test. (A) The instant HR responses of an individual mouse showed clearly two distinct stages: stage-I (red) and stage-II (blue) during the contextual habituation and retention tests. Upper panel: contextual habituation; Middle panel: 1-hr contextual retention test; Lower panel: 1-day contextual retention test. The freezing responses were plotted on top of the instant HR; freezing state, red bar; non-freezing state, blue bar. The blue vertical line indicates the start of stage-I; the red vertical line indicates the end of stage-I; the green vertical line indicates the end of the contextual retention test. (B) Poincaré plots of the same mouse’s R-R intervals of 1-min duration in prior (grey line), stage-I (red line) and stage-II (blue line). The prior was a 3-min period before contextual habituation or retention. Upper panel: contextual habituation; Middle panel: 1-hr contextual retention test; Lower panel: 1-day contextual retention test. (C-D) The HR and CV of prior, stage-I and stage-II during 1-hr contextual retention. Error bars, s.e.m.; n = 11; *P<0.05, ***P<0.001, one-way repeated measures ANOVA and Tukey *post hoc* test.

To understand how HRV and HR change during memory retrieval, we conducted contextual fear retention tests, first with 1-hr retention test, and then followed by 1-day retention test. During the 1-hr contextual retention test (15-min), the instant HR showed a 2-stage profile (Figure 4A). The first stage was characterized as a 'plateau' (stage-I) during which HR was increased (HR  = 748 bpm) but HRV was extremely small (CV  = 0.49%), whereas the second stage was characterized as lifted instant HR but slightly increased CV of instant HR. The Poincaré plot of R-R intervals of habituation session showed the existence of two distinct HRV stages (Figure 4B, top row), but with a shorter stage-I duration and larger variability in stage-II. During contextual fear recall, the HR significantly increased when mice were introduced into the fearful context in 1-hr contextual retention or 1-day contextual retention test (Figure 4A). Moreover, HRV also showed greater reduction during 1-hr or 1-day contextual retention tests. We observed that consolidation of short-term contextual fear memory into long-term memory was associated with an increase in the HRV stage-I duration (Figure 4A) as well as more tightly regulated HRV in the stage-II (Figure 4B). Our quantitative analyses show that HR did not show significant difference between these two stages during contextual recall although both were significantly higher than the base rate (Figure 4C, *P*<0.001). However, HRV, as measured by the CV of instant HR, revealed the significant difference between the two stages as well as to that of the baseline prior to recall (Figure 4D, *P*<0.05, *P*<0.001).

**Figure 5:**
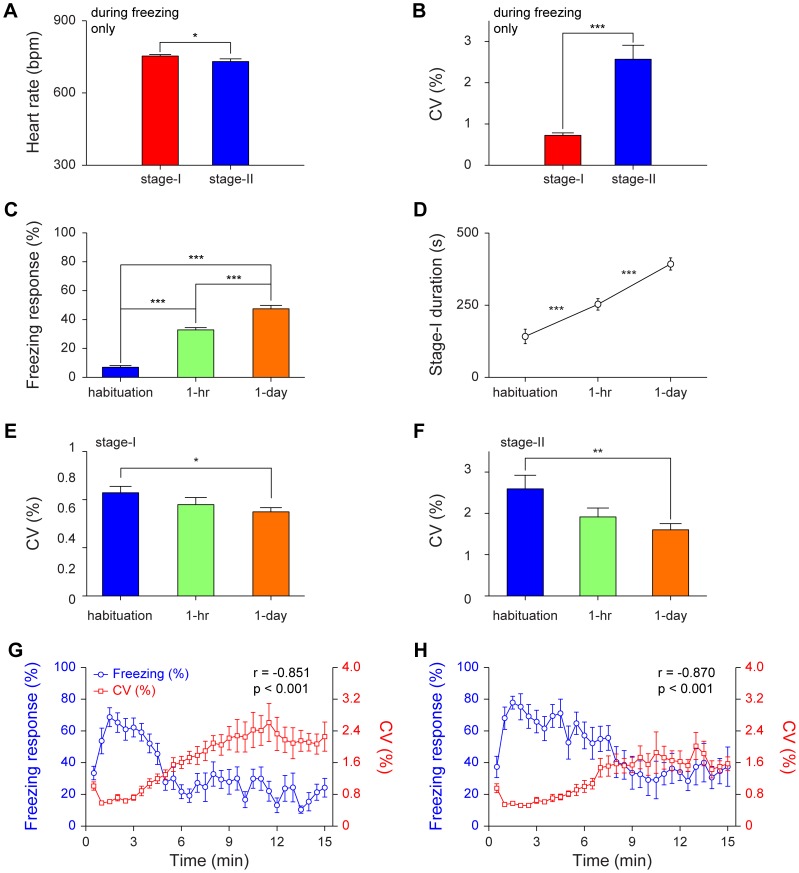
Changes in heart rate and heart rate variability in relationships with freezing behavior during contextual recall. (A–B) The HR and CV of stage-I and stage-II freezing period in 1-hr contextual retention test. n = 11; *P<0.05, ***P<0.001, paired *t* test. (C) The freezing responses during contextual habituation, contextual 1-hr retention and contextual 1-day retention. n = 8; ***P<0.001, one-way repeated measures ANOVA and Tukey *post hoc* test. (D) The duration of stage-I during contextual habituation, 1-hr contextual retention and 1-day contextual retention. n = 8; ***P<0.001, one-way repeated measures ANOVA and Tukey *post hoc* test. (E–F) The CV of stage-I and stage-II during contextual habituation, 1-hr contextual retention and 1-day contextual retention. n = 8; *P<0.05, **P<0.01, one-way repeated measures ANOVA and Tukey *post hoc* test. (G) The averaged freezing responses in eleven mice were anti-correlated with the averaged CV of instant HR in 1-hr contextual retention (r = –0.851, *P*<0.001). (H) The averaged freezing responses in eight mice were also anti-correlated with the averaged CV of instant HR in 1-day contextual retention (r = –0.870, *P*<0.001). All data are plotted as mean ± s.e.m. (error bars).

To examine relationship between heart physiology and freezing behavior, we investigated the HR and the CV of instant HR during freezing moments. During the freezing period, the HR in stage-I was slightly but significantly higher than in stage-II (Figure 5A, *P*<0.05). In the meantime, HRV in stage-I was much lower than in stage-II (Figure 5B, *P*<0.001). Behaviorally, there were significant differences in freezing responses between contextual habituation, 1-hr contextual retention, and 1-day contextual retention tests (Figure 5C, *P*<0.001). We found that the durations of stage-I represented a highly correlated physiological indicator for fear memory formation (Figure 5D, *P*<0.001). Moreover, the CV of stage-I became tighter as the mice transit from habituation to 1-hr retention test and to 1-day retention test (Figure 5E, *P*<0.05). Similarly, the CV of stage-II showed the same trend (Figure 5F, *P*<0.01). The CV of 3-min baseline prior to 1-hr or 1-day recall showed no statistical significance from that of prior to habituation (P>0.05). The fear conditioning did not seem to have effect on basal HR dynamics.

As a group, averaged freezing percentages during the contextual retention tests were nicely anti-correlated with the averaged CV of instant HR in 1-hr retention (Figure 5G, r = –0.851, *P*<0.001)and 1-day retention (Figure 5H, r = –0.870, *P*<0.001). It should be noted that in 1-hr contextual retention test, the crossing point for freezing and CV of instant HR in the plot was at around 5-min, but in 1-day contextual retention test, the crossing point was at around 8-min. This again indicates that HRV changes (stage-I phenomenon), just like freezing measurement, is useful for assessing fear memory formation.

### HRV reduction during auditory cued fear memory recall

**Figure 6:**
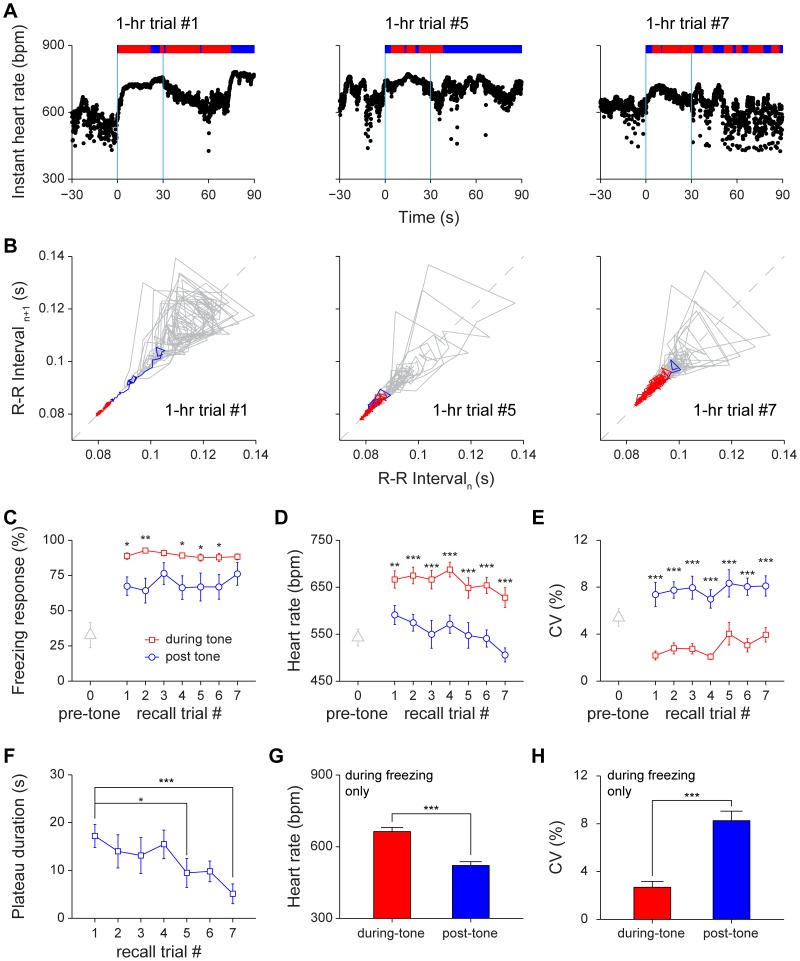
Changes of heart rate and heart rate variability in 1-hr cued fear retention tests. (A) Instant HR responses of an individual mouse during three recall trials, trial#1, trial#5 and trial#7. The freezing responses were plotted on top of the instant HR; freezing state, red bar; non-freezing state, blue bar. The blue vertical lines indicate the onset and offset of the tone (30 seconds). (B) Poincaré plots of the same mouse’s R-R intervals of 30-sec pre-tone (grey line) and 30-sec during-tone (blue line and red line) in trial #1, trial #5 and trial #7. The blue line indicates the rising phase of instant HR, which was defined as a period from the onset of tone to the time when HRV reached the stage-I plateau. (C–E) The freezing responses, HR and CV of pre-tone, during-tone and post-tone during 1-hr auditory cued retention. n = 11; *P<0.05, **P<0.01, ***P<0.001, paired *t* test. (F) The stage-I plateau durations during 1-hr auditory cued retention. n = 11; *P<0.05, ***P<0.001, one-way repeated measures ANOVA and Dunnett’s multiple comparisons test. Dunnett’s multiple comparisons test comparing with recall trial #1 showed that extinction effect reached significant difference at trial #5. (G–H) The HR and CV of the during-tone and post-tone freezing period in 1-hr auditory cued retention test. n = 11; ***P<0.001, paired *t* test. All data are plotted as mean ± s.e.m. (error bars).

To assess how the retrieval of cued fear memory changes HR and HRV (with the minimal effect or interference from environmental effects), we used the home cage situation for cued recall so that the conditioned tone was played as mice remained in their home cages. In 1-hr cued retention test, the instant HR increased upon hearing the tone (Figure 6A). Yet we found that HRV was more sensitive than HR itself under multiple CS retrieval protocol (CS tone was played seven times with 3–5 min randomized intervals) (Figure 6A). The Poincaré plot showed increase in heartbeat regularity during the 30-sec conditioned tone period (see red segments in Figure 6B).

Behaviorally, since the cued retention test was conducted in the home cage, mice in the pre-tone phase (90 seconds) showed more resting behavior or immobility (in comparison to more active exploratory behavior in a new environment), which caused the pre-tone freezing was higher (32.7±8.9%). Still mice were able to show significant freezing responses during cued retention test (89.3±1.6%, 30-sec tone period, *P*<0.001). We further counted freezing behavior during the 60-sec post-tone period and found that it was slightly but significantly lower than that of 30 sec during-tone period (Figure 6C, *P*<0.05, *P*<0.01). As expected, the HR significantly increased during 30-sec tone period and remained high over repeated tone trials (*P*<0.01). During the 60-sec post-tone period, the heart rate seemed to drop significantly over repeated recall trials during the short-term retention test (Figure 6D, *P*<0.01, *P*<0.001).

During 1-hr cued recall, we observed that HRV, as measured by CV, significantly decreased during tone (Red curve in Figure 6E, *P*<0.05), but quickly become more variable after the 30-sec tone period (Blue curve in Figure 6E, *P*<0.001). Although measurements of both the during-tone and post-tone freezing responses did not show significant changes across trials (*P*>0.05), HRV analysis revealed that heartbeat regularity and the stage-I plateau durations were sensitive enough to show the noticeable extinction effect (Figure 6B and 6F). The stage-I plateau duration showed a statistically significant decreasing trend (Figure 6F, *P*<0.05, *P*<0.01).

To provide further understanding of the relationship between fear memory, freezing, and HR, we examined whether the HR and HRV differs during the freezing states between the tone period and post-tone period. Our analysis showed the different physiological statuses under these two freezing phases. During the freezing state of 30-sec tone period, HR was significantly higher than that of the post-tone freezing period (Figure 6G, *P*<0.001). Moreover, CV during the 30-sec tone-induced freezing state was significantly lower than that of the post-tone freezing state (Figure 6H, *P*<0.001). This strongly suggests that HRV dynamics is a sensitive physiological indicator for measuring subtle emotional change in the during-tone period as well as the post-tone period.

**Figure 7:**
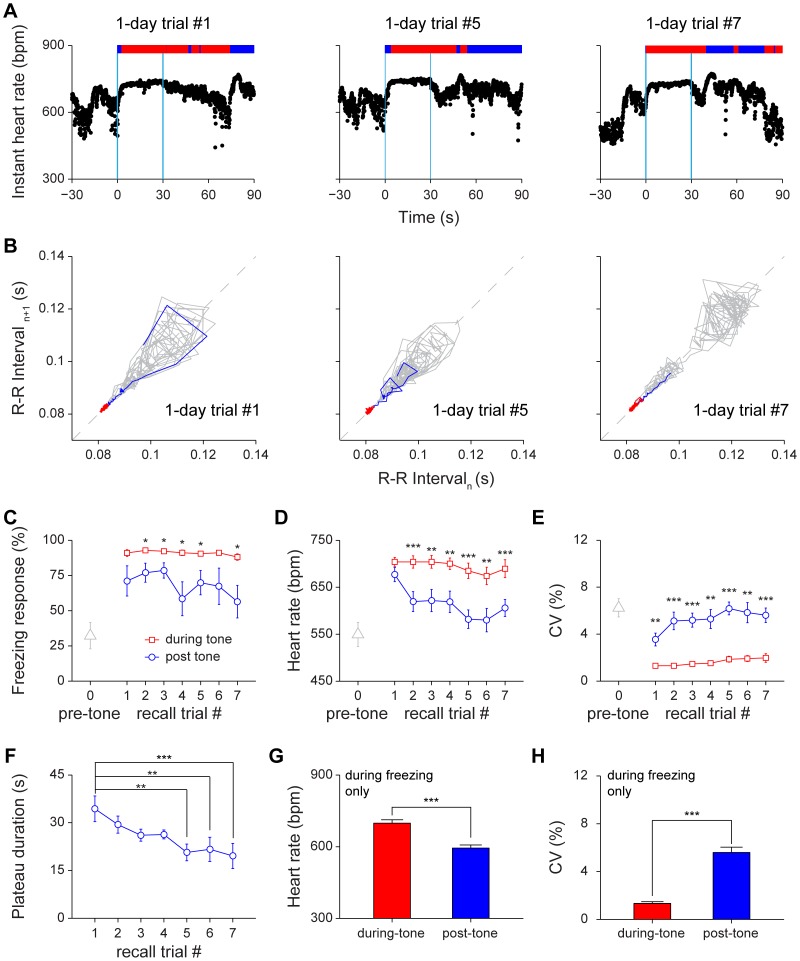
Changes of heart rate and heart rate variability during 1-day cued fear retention tests. (A) Instant HR responses of the same mouse during three recall trials, trial#1, trial#5 and trial#7. The freezing responses were plotted on top of the instant HR; freezing state, red bar; non-freezing state, blue bar. The blue vertical lines indicate the onset and offset of the tone (30 seconds). (B) Poincaré plots of the same mouse’s R-R intervals of 30-sec pre-tone (grey line) and 30-sec during-tone (blue line and red line) in trial #1, trial #5 and trial #7. The blue line indicates the rising phase of instant HR, which was defined as a period from the onset of tone to the time when HRV reached the stage-I plateau. (C–E) The freezing responses, HR and CV of pre-tone, during-tone and post-tone during 1-day auditory cued retention. n = 8; *P<0.05, **P<0.01, ***P<0.001, paired *t* test. (F) The stage-I plateau durations during 1-day auditory cued retention. n = 8; **P<0.01, ***P<0.001, one-way repeated measures ANOVA and Dunnett’s multiple comparisons test. Dunnett’s multiple comparisons test comparing with recall trial #1 showed that extinction effect reached significant difference at trial #5. (G–H) The HR and CV of the during-tone and post-tone freezing period in 1-day auditory cued retention test. n = 8; ***P<0.001, paired *t* test. All data are plotted as mean ± s.e.m. (error bars).

Next, we asked how expression of long-term fear memory affected HR and HRV by conducting 1-day cued retention test. Similar to short-term cued recall, tone induced changes in HRV and HR (Figure 7A). The Poincaré plot showed more stable conditioned responses during tone recall (Figure 7B). Freezing behavior during the 30-sec tone period was significantly higher than that of post-tone period in most trials and became much more consistent (Figure 7C, *P*<0.05). The during-tone HR was significantly higher than post-tone HR, (Figure 7D, *P*<0.01, *P*<0.001). Also, the during-tone HRV was significantly lower than post-tone HRV (Figure 7E, *P*<0.01, *P*<0.001). Although the heartbeat regularity during tone was much more stable throughout trials in comparison to short-term retention test, statistical analysis shows that the extinction effect, as indicated by reduction in the stage-I plateau duration, was clearly noticeable (Figure 7F, *P*<0.01, *P*<0.001). Consistent with those observed in 1-hr cued retention tests, the HR of during-tone freezing period was also significantly higher than that of post-tone freezing state (Figure 7G, *P*<0.001). Moreover, the during-tone HRV was significantly lower than the post-tone HRV in freezing (Figure 7H, *P*<0.001).

**Figure 8:**
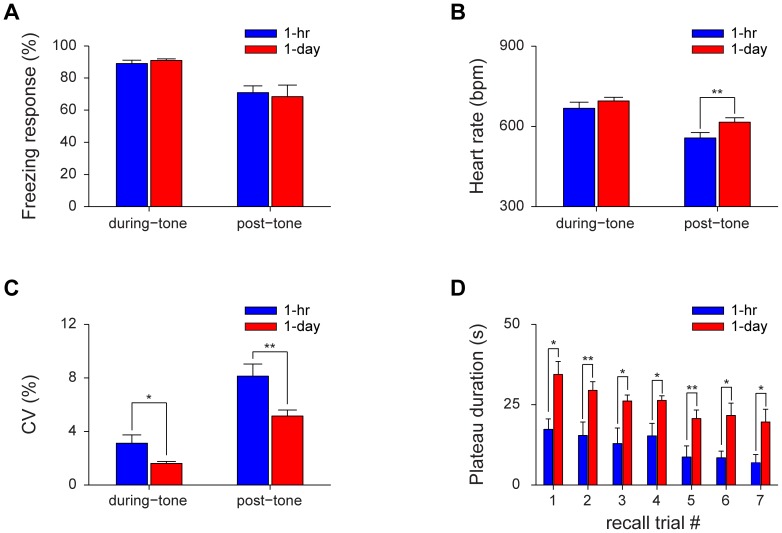
Comparison of heart rates and heart rate variabilities between short-term and long-term cued fear memories. (A) There was no difference in the total amount of freezing during either 30-sec tone periods or 90-sec post-tone periods between 1-hr and 1-day retention test. (B) The post-tone HR of 1-day auditory cued retention test was significantly higher than that of 1-hr retention test. n = 8; **P<0.01, paired *t* test. (C) The CV of during-tone and post-tone periods in 1-day auditory cued retention test was greatly reduced compared with 1-hr retention test. n = 8; *P<0.05, **P<0.01, paired *t* test. (D) The plateau durations during 1-day auditory cued retention were significantly longer than that during 1-hr retention. n = 8; *P<0.05, **P<0.01, paired *t* test. All data are plotted as mean ± s.e.m. (error bars).

Finally, we asked how HR and HRV changes correlated with the transition and consolidation of fear memory from short-term to long-term memory. We found that there was no difference in the total amount of freezing during 30-sec tone periods between 1-hr and 1-day cued recall (Figure 8A). There was also no difference in the amount of freezing during the post-tone period between 1-hr and 1-day retention tests. This suggests that freezing scoring method is not ideal for measuring fear memories in the home cage environment. On the other hand, while the during-tone HR showed no significant difference between 1-hr and 1-day retention tests, the post-tone HR of 1-day retention was significantly higher than 1-hr retention (Figure 8B, *P*<0.01). Furthermore, compared with 1-hr retention test, both the CV of during-tone and post-tone periods were greatly reduced (Figure 8C, *P*<0.05, *P*<0.01). Most importantly, the durations of HRV stage-I plateau at 1-day retention tests were significantly longer than those of 1-hr retention tests (Figure 8D, *P*<0.05, *P*<0.01). The reduced CV and longer plateau duration at the time of 1-day cued memory recall show that the HRV is a valuable parameter for reflecting a more stable consolidated fear memory.

## Discussion

Our above experiments described HR and HRV dynamics in mice during both acquisition and retention sessions of contextual and cued auditory fear conditioning. Traditionally, HR has been used as a physiological readout of a conditioned response [16], [23]. Our finding here suggests that while HR itself is an important parameter, HRV dynamics can serve as a sensitive measurement for reflecting emotional states when recalling short-term and long-term contextual and cued fear memories.

Because of the well-defined cues and memory produced by fear conditioning paradigms, extensive efforts have been invested in electrophysiological analyses of neural changes in the brain [5], [24]–[30]. Because identification of neural responses of recorded neurons *in vivo* requires multiple CS/US pairings to aid the peri-event spike raster or histogram analyses [20], [27], [31]–[33], our study examined the effects of CS/US pairing on HR and HRV. We further systematically examined the relationships between HR/HRV and fear memory recall. We found that as a group, the most significant increase in HR occurred upon the first foot shock, whereas reduction in HRV and increase in freezing responses exhibited most significant change at the second CS/US pairing. This indicates that change in HR is a good physiological parameter, but did not fully capture emotional changes related with fear learning behavior such as freezing. HR can be easily influenced by additional physical states (such as running) that are not necessarily related to emotion or fearfulness. On the other hand, while increased exploratory behaviors in novel environment were associated with elevated HR, it is the HRV stage-I duration that was sensitive to and correlated with environmental habituation (Fig 2C). This suggests that the dynamics of HRV could be more informative for evaluating novelty and emotional changes in mice.

In contextual retention tests, both the HRV stage-I and stage-II showed elevated HR. However, the variability of HR in stage-I is significantly smaller than that in stage-II, suggesting that these two stages are physiologically different. These two stages could not be readily distinguished using freezing scores. In the literature, contextual retention tests were conducted typically for a period of 5-minutes. Our current finding provides a physiological basis or rationale for this time duration used for measuring freezing. Furthermore, our results suggest that HRV parameter reflects well on the consolidation or transition of new contextual short-term memory to long-term contextual memory. The duration of HRV stage-I period exhibited in 1-day contextual recall test was longer than that in 1-hr contextual recall test (393±21 seconds vs. 253±20 seconds, *P*<0.001). This can provide another means to confirm the transition from short-term memories into long-term memories.

In cued fear memory recall, we used the 30-sec tone to trigger freezing behavior. It is very interesting to note that the heightened heart beat regularity period (HRV stage-I) in 1-day retention was much longer than that of 1-hr retention (Figure 8D). The gradual shortening of HRV stage-I plateau duration over repeated tone recall trials (Figure 6F and Figure 7F) demonstrated the extinction effects which were not so obvious based on the freezing level (Figure 6C and Figure 7C). This further suggests that HRV is a highly sensitive physiological indicator for measuring emotional learning and memory.

In summary, we have systematically characterized HR and HRV in both short-term and long-term fear conditioning. Our results suggest that HRV can serve as an important physiological readout for assessing both the initial learning phase as well as the short-term to long-term fear memories recall. Such dynamic physiological readout, once coupled with large-scale neural *in vivo* recording approaches [20], [34], can be valuable for deeper understanding of various trace components of fear memory engram in the mouse brain.

## Materials and Methods

### Ethics statement

All animal work described in the study was conducted in accordance with the National Institutes of Health guidelines, and approved by the Institutional Animal Care and Use Committee of Georgia Regents University and BBRI.

### Subjects

A total of 11 adult male C57BL/6J mice were used for the experiments. Electrocardiogram (ECG) measurements were performed on all 11 mice. To classify different behavioral states, hippocampal local field potentials (LFPs) were recorded from five of them. Mice were individually housed on a 12:12-h light-dark cycle (Lights on at 8:00 a.m.) and had access to food and water *ad libitum*.

### Surgeries

One week before surgery, mice (5–6 months old) were transferred from the standard cages to customized home cages (19” in diameter, 16.5” in height). The home cages were changed and cleaned daily. On the day of surgery, the mice were deeply anesthetized with Ketamine/Domitor (60/0.5 mg/kg, i.p.). For ECG surgery, a single pair of insulated electrodes was placed subcutaneously from the back of the neck to the chest in the lead II configuration [35]. Using non-absorbable suture, the positive electrode, which was placed in the left abdomen below the heart, was anchored to the underlying peritoneal tissue. The negative electrode, which was placed in the right upper chest, was anchored to the pectoral muscle (Figure 1A). The incisions of the skin were sutured. For hippocampal LFP recording, a 32-channel (a bundle of 8 tetrodes), screw-driven electrode array was implanted toward unilateral dorsal hippocampal CA1 region (2.3 mm posterior and 2.0 mm lateral to bregma, 0.8 mm ventral to the brain surface), similar to that previously described [34]. Antisedan (2.5 mg/kg, i.p.) was applied to awaken the mice. After surgery mice were allowed to recover for a week. Five mice were implanted both the ECG leads and the electrode array.

### Electrocardiogram (ECG) and local field potential (LFP) Recording

All electrodes were pre-attached to a miniature connector (Omnetics). The ECG electrodes, as well as the hippocampal electrode array, were then connected to Multichannel Acquisition Processor system (Plexon Inc., Dallas, TX). ECG and LFP signals (filtered at 0.7–300 Hz, digitized at 5 kHz) were recorded by using Plexon Sort Client software. Mice behaviors were monitored and recorded simultaneously by using the Plexon CinePlex video recording and tracking system. The electrode array was advanced slowly, in daily increments of about 70–140 µm, until the tips of the electrodes reached the CA1 pyramidal cell layer, as deduced from an assessment of high-frequency ripples.

### Behavioral Paradigm

Mice were handled twice a day (5 minutes each session) for 5 days to minimize the potential stress of human interaction.

#### Novel Environment Exposure

Either a triangular (15” each side, 15” in height) or round chamber (11” in diameter, 15” in height) was used as the novel environment. Mice were transferred from home cage to the novel chamber for a 15-min free exploration. The exploration was repeated in the same environment for three times with 30-min intervals.

#### Fear Conditioning Task

The fear conditioning chamber was a square chamber (10’’×10”×15”) made of plastic boards with a 24-bar inescapable shock grid floor. Mice behaviors in the chamber were recorded by using the Plexon CinePlex video recording and tracking system. Freezing response was defined as absence of body movement except for respiration. Based on the recorded videos, freezing responses were measured frame by frame. The temporal resolution of the video is 30 frames per second. Mice were considered to be freezing if no movement was observed for at least 2 seconds.

On the training day, the mouse was exposed to 30-sec, 85 dB tone seven times in the home cage for tone habituation. Then the mouse was introduced to the shock chamber for a 15-min contextual habituation. The mouse was brought back to the home cage for a 30-min rest before cued fear conditioning began. During cued fear conditioning, the mouse was allowed to be habituated to the shock chamber for another 15 minutes to minimize the effect of novel environment on HR. Then the conditioned stimulus (tone, 30-sec, 85 dB, 5000 Hz) was given and then co-terminated with the unconditioned stimulus (a continuous 2-sec mild foot shock at 0.75 mA). This CS-US pairing was repeated for seven times, with 3–5 min randomized intervals between trials. The mouse was then brought back to the home cage for 1-hour sleep/rest. The during-tone freezing responses were measured for 28 seconds prior to 2-sec foot shock.

After a 1-hr rest, contextual and cued memory retention tests were conducted. During 15-min contextual memory retention test, the mouse was placed back to the shock chamber with the exact contextual settings, but without tone and foot shock. The cued memory retention test was conducted in the home cage. A 30-sec tone was played, repeated seven times with 3–5 min randomized intervals. The pre-tone, during-tone and post-tone freezing responses were measured for 90 seconds, 30 seconds and 60 seconds respectively. After the completion of all retention tests, the mouse was allowed to rest for 30 minutes. Then the mouse was placed back to the shock chamber for retraining (one CS-US pairing), and then returned to the home cage to rest. One day later, the mouse was subjected to a 15-min contextual retention test, and then a cued retention test (30-sec tone repeated seven times).

### Data analysis

#### Behavioral states classification

Four different behavioral states, i.e., active wakefulness (AW), quiet wakefulness (QW), rapid eye movement (REM) sleep, and slow wave sleep (SWS) were classified by a combination of CA1 LFPs and behavioral criteria [36], [37]. The four behavioral states were measured in home cages. AW was characterized by occurrence of gross body movements; some of the periods were accompanied by theta oscillations. QW was characterized by variable low amplitude LFPs, without gross body movements, but with the head up and eyes open; in this state, LFPs were devoid of theta oscillations, but intermittent ripple oscillations were detected. REM sleep was characterized by stillness, eyes closed and prominent theta oscillations. SWS was characterized by stillness, eyes closed and large irregular activity in LFPs, with intermittent ripple oscillations.

#### Heart rate variability (HRV) analysis

The discrete timestamps of R-wave maxima were obtained by peak detection algorithm. The R-R intervals were converted into instant HR (beats per minute). For stage-I detection, the threshold method combined with a sliding-window technique was used. The threshold to determine the start and end of the ‘plateau’ was set as follows: first, for each mouse, choose a 180-sec active wakefulness period as baseline; second, vector 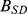 was calculated as a set of standard deviation of instant HR during baseline period using a 30-point-width bin, sliding at 1-point time resolution; third, the threshold 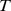 was defined as: 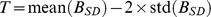. Beginning with the onset of the stimulus, the standard deviation of instant HR was calculated using a 30-point-width bin, sliding at 1-point time resolution. The time of the first point below the threshold 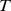 was selected as the start of the ‘plateau’. The end of the ‘plateau’ was defined as the time of the first point of the successive points above the threshold 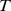 for 5 seconds. The variability of R-R intervals was graphically described as Poincaré plot. Each heart beat interval, R-R interval_n_ was plotted on the X-axis against the subsequent heart beat interval, R-R interval_n+1_ on the Y-axis. The coefficient of variation (CV) of instant HR was based on the formula: 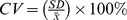, where 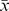 (mean) and 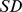 (standard deviation) were the 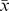 and 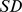 of instant HR.

#### Statistical analysis

For the comparisons of multiple means, one-way repeated measures ANOVA and Tukey *post hoc* tests were conducted to assess the difference of means. Dunnett’s multiple comparisons test was also performed to compare the means at individual time points to the control (first trial). Differences between two means were assessed with paired *t* test. Data are presented as mean ± s.e.m. Differences were considered significant if *P* values were <0.05.
